# 

**Published:** 1966-10

**Authors:** 


NATIONAL LIBRARY OF MEDICINE

RECD. DEC 20 1966

SHELVED IN RR: YES........NO X

CALL NO.: W1 BR323

COPY NO. 1 2 3 4 5

CHECKED K

INCORPORATING THE MEDICAL JOURNAL OF THE SOUTH WEST

Vol 81 (iv) No 302

